# Dataset of numerically-generated interfaces of Newtonian jets in CIJ regime

**DOI:** 10.1016/j.dib.2022.108215

**Published:** 2022-04-27

**Authors:** Guillaume Maîtrejean, Adeline Samson, Denis C.D. Roux

**Affiliations:** a Universit Grenoble Alpes, CNRS, Grenoble INP (Institut of Engineering Universit Grenoble Alpes), LRP, Grenoble 38000, France; b Universit Grenoble Alpes, CNRS, Grenoble INP (Institut of Engineering Universit Grenoble Alpes), LJK, Grenoble 38000, France

**Keywords:** Rayleigh-Plateau instability, Jet of fluid, Drop, Continuous Ink Jet

## Abstract

The so-called Rayleigh-Plateau instability of fluid jets has been widely studied and is extensively used in the Continuous InkJet (CIJ) printing process. The present dataset contains the numerically-generated interfaces of Newtonian fluids jets in CIJ jetting conditions for low to moderately high stimulation amplitudes. We used Basilisk, an open-source Computational Fluid Dynamics (CFD) software specialized in multiphase flow to compute thousands of jets of fluids for Reynolds numbers ranging from 100 to 1000. The dataset gives raw data of CFD simulations liquid-air interfaces, for each Reynolds – stimulation amplitude pair. The present 10 GB dataset contains ≈110000 interfaces which allows to use novel machine learning and deep-learning approaches to explore jet morphologies evolution that can’t be addressed with the classical Rayleigh’s theory.

## Specifications Table

**Table:** 

Subject	Hydrodynamics
Specific subject area	Multiphase Flow, Jets of fluids, Continuous InkJet
Type of data	Text Files
How data were acquired	Numerical simulation using Basilisk software
Data format	Raw
Parameters for data collection	Jets are axisymmetric, with dimensionless radius of 1 and dimensionless inlet velocity of 1. The periodic amplitude disturbance $u_{0}$ that will trigger and control the Rayleigh-Plateau instability writes
	$u_{0}=\left\{1+\delta \sin(2\pi f_{r}t),0\right\},\;\;\;\text{(1)}$
	where the dimensionless frequency is $f_{r}=\frac{1}{7}$ and δ is the stimulation amplitude which ranges from 1 to 3.5% of the velocity inlet with a 0.5% interval. The fluids viscosities range from Reynolds 100 to 1000 with an interval of 5. The surface tension is fixed so as the Weber number is We=600. The density and viscosity ratios between the gaz and liquid phases are fixed to 1000 and 500, respectively.
Description of data collection	The dataset has been numerically-generated using the open-source software Basilisk [1] on the GRICAD infrastructure (https://gricad.univ-grenoble-alpes.fr)
Data source location	Grenoble Alpes Université, Grenoble, France
Data accessibility	https://data.mendeley.com/datasets/3ds9h73pnv/1

**Value of the Data**

• The present dataset gives the interfaces of jetted fluid for both a large range of Reynold numbers and disturbance amplitudes. These interfaces, that can’t be analytically retrieved, are generated solving the full Navier-Stokes equation which are computationally intensive simulations.
• The data can be useful for engineers and researchers who work in the fluid jetting research area with particular focus on CIJ.
• The information contained in the present dataset can support either fundamental research on jetted fluids and drops by comparing analytical development to numerical simulations or applied CIJ research by developing further the process.
• By using machine learning and more specifically deep learning approaches, data may give a better insight of the morphology of CIJ jets – i.e. breakup-length, satellite regime, drop shape, etc – and allow to further improve the CIJ process or even use it for new application fields.

## 1 Data Description

**Fig. 1:**
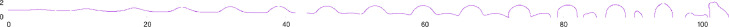
Interface of the jetted fluid plotted using Gnuplot [2], generated with Re=975, Amp=0.035 and saved at Time=102.06.

The data is organized as follows: for each Reynolds number and stimulation amplitude data files are in a folder named Re_Amp, with Re the Reynolds number ranging from 100 to 1000 and Amp the stimulation amplitude ranging from 0.01 to 0.035. In each folder, i.e. for every Reynolds and stimulation amplitude pair, 101 raw text files are provided named *interfaces_Re_Amp_Time.dat* where Time refer to the simulation time at which the interface has been saved (see next section for a more detailed description of the simulation). The data in the interface files are in Gnuplot-style [2] format: the interface is described by segments of 2 points, with an x and y position, separated by a line break. Fig. 1 illustrates the generated interface (Re=975, Amp=0.035 at Time=102.06) plotted using Gnuplot.

Along with the interface files, in each folder is *coefficients.csv* file which contains information for the first 10 drops for every Time.

The first three columns, Reynolds, Amplitude and Time are explicit; for every drop, six columns containing the barycenter, width, height, Feret diameter, area and volume of the drop are computed during the simulation and added to the file as illustrated 1. Note that the computation of both the area and volume account for the model axisymmetry.

**Table 1:** The 15 first columns and the 5 first rows for Re=100 and Amp=0.01 in the coefficients file.

Reynolds	Amplitude	Time	Barycenter	Width	Height	Feret	Area	Volume	Barycentre.1	Width.1	Height.1	Feret.1	Area.1	Volume.1
100	0.01	378.14	168.35	335.12	1.68	199.52	2127.87	1093.64	339.98	3.42	1.74	1.96	37.53	21.56
100	0.01	378.21	168.39	335.19	1.68	199.55	2128.24	1093.85	340.04	3.42	1.74	1.96	37.53	21.56
100	0.01	378.28	168.42	335.25	1.68	199.59	2128.60	1094.07	340.11	3.41	1.74	1.96	37.52	21.56
100	0.01	378.35	168.45	335.31	1.68	199.63	2129.02	1094.29	340.18	3.41	1.74	1.96	37.52	21.56
100	0.01	378.42	168.49	335.39	1.70	197.83	2129.35	1094.51	340.24	3.41	1.74	1.96	37.52	21.56

**Note:** As pictured on Fig. 2, the main jet is always considered as the first drop and thus numbered *drop 0*. The drops are numbered from the inlet (left) to the outlet (right) and the drop numbering is not preserved over the simulation and changes when a new drop is generated. A maximum of 10 drops are kept and *NA* values are added where no data is available, i.e. when less than 10 drops are present.

**Fig. 2:**
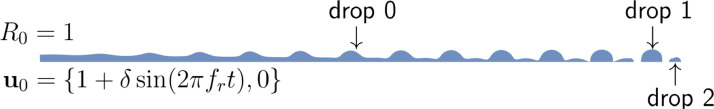
Example of an axisymmetric numerically-generated jet using Basilisk software.

A Jupyter notebook with minimal working examples using part of the present dataset can be found at https://gricad-gitlab.univ-grenoble-alpes.fr/maitrejg/numerically-generated-interfaces-of-newtonian-jets-in-cij-regime.

## 2 Experimental Design, Materials and Methods

### 2.1 Governing equations

The dataset has been numerically-generated using Basilisk software [1] which is dedicated to solving partial derivative equations. It uses a tree data structure (quadtree in 2D and octree in 3D) that allows to refine locally and dynamically the mesh based on automatic or user-defined criteria [3]. In the present case it solves the multiphase, unsteady and incompressible Navier-Stokes equations 

(2) $$\partial_{t}u+\nabla \cdot \left(u⊗u\right)=\frac{1}{\rho }\left[-\nabla p+\nabla \cdot (2\mu D)\right]$$

and

(3) ∇·u=0

with u the velocity field, ρ the density of the considered phase, D the deformation tensor such as $D=[\nabla u+{\left(\nabla u\right)}^{T}]/2$ and μ the dynamic viscosity.

The interface between the fluids is tracked with a Volume-Of-Fluid (VOF) method [4]. At the interface the term

(4) $$\frac{1}{\rho }\sigma \kappa \nabla f$$

is also added to the right-hand side of Eq. (2) to account for the surface tension effect, with σ the (constant) surface tension, κ the interface mean curvature and f the volume fraction of fluids describing the interface.

The model is axisymmetric (see Fig. 2) with a velocity boundary condition $u_{0}$ on the liquid phase with a periodic amplitude disturbance that will trigger and control the Rayleigh-Plateau instability

(5) $$u_{0}=\left\{1+\delta \sin(2\pi f_{r}t),0\right\},$$

with δ the disturbance amplitude, $f_{r}$ the frequency of the perturbation and t the simulation time. The frequency is fixed to $f_{r}=1/7$ for all jets.

An outflow boundary condition is imposed on all the remaining boundaries.

The initial radius $R_{0}$ is set to 1 and both the density and the viscosity ratio of the liquid-gas system are fixed to 1000 and 500, respectively. The Reynolds Number

(6) $$Re=\frac{\rho_{l}u_{0}^{t=0}R_{0}}{\eta_{l}}$$

is directly related to the inverse of the Newtonian viscosity (the subscript l stands for liquid) as $\rho_{l}$, $R_{0}$ and $u_{0}^{t=0}$ are set to 1 for all jets.

### 2.2 Meshing strategy and convergence

Basilisk provides a powerful Automatic Mesh Refinement (AMR) strategy based on the use of quadtrees (in 2D). The domain is a square of dimension 512 and the mesh is refined locally and dynamically based on a wavelet-estimated discretization error [5]: a user-defined list of fields is analysed and the mesh is refined/coarsened based on a user-defined error criteria (one per field). In the present simulation, the adaptation is based on both the phase fraction f and the velocity fields u with $10^{-4}$ and $10^{-3}$ error criteria, respectively, with a user-specified refinement level n, such as the element size $\Delta_{x}$ can be as small as $\Delta_{x}=\frac{512}{2^{n}}$ in the most refined zone.

To assess the most efficient meshing approach, three strategies are compared in term of accuracy and performance:

1. An automatic refinement up to the maximum level is forced around the interface, i.e. in zones where the fluid fraction is between zero and one. This approach is similar to what can be performed with Gerris software [6], the Basilisk’s predecessor (and done in [7] for example).
2. Taking advantage of the adaptive wavelet algorithm of the Basilisk toolbox to refine/coarsen where it is needed, no matter the liquid fraction value. The adaptation is based on both the phase fraction f and the velocity fields u with $10^{-4}$ and $10^{-3}$ error criteria, respectively.
3. Mixing the above two strategies by forcing the finest mesh close the interface and adapting elsewhere is needed. This strategy will be considered as a reference as it should be the most accurate.

As one can expect, the meshing strategy has a great impact on the number of cells and, consequently, on the computation time. Tab. 2 gives the CPU time spent for each strategy using 4 cores on the same CPU (Intel E5-2670). Strategy 1 is the fastest while the other two strategies are more expensive as the surrounding air is also partly refined although not having a great influence on the jet morphology.

**Table 2:** Calculation time spent on the same test case for the 3 meshing strategies

	CPU time (s)
Strategy 1	9500
Strategy 2	42000
Strategy 3	49000

**Fig. 3:**
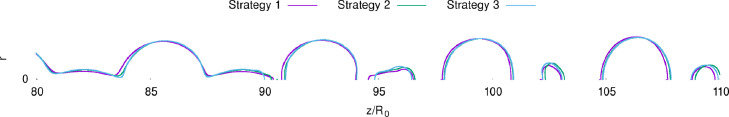
Interfaces of the jet at the breakup, depending on the meshing strategy. Re=500 at t=128.

When comparing the jet morphologies at the breakup and the same simulation time of t=128 and for Re=500 (Fig. 3), the 3 strategies give a very close morphology, with the best agreement between strategies 2 and 3. Hereafter, strategy 2 will be used for all the simulations as it is slightly more accurate in term of interface shape than strategy 1 and less computationally expensive than the last strategy.

**Fig. 4:**
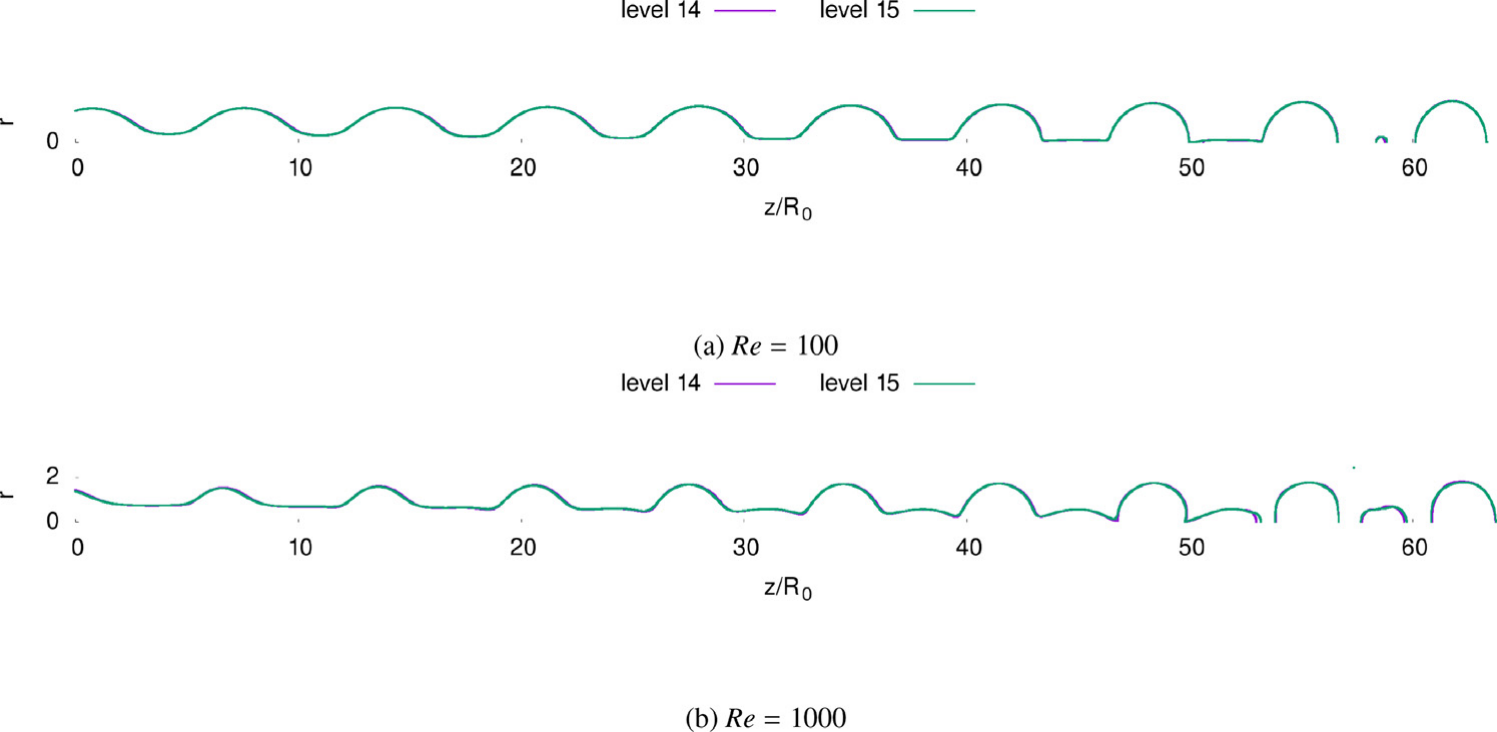
Fluid-air interfaces obtained at refinement levels 14 and 15 for both Re=100 (a) and Re=1000 (b).

Using the meshing strategy 2, a convergence study is then performed and it has been found that the converged refinement level is 15: as pictured Figure 4, the interfaces obtained with a refinement level of 14 are almost identical to those obtained with a refinement of 15.

**Fig. 5:**
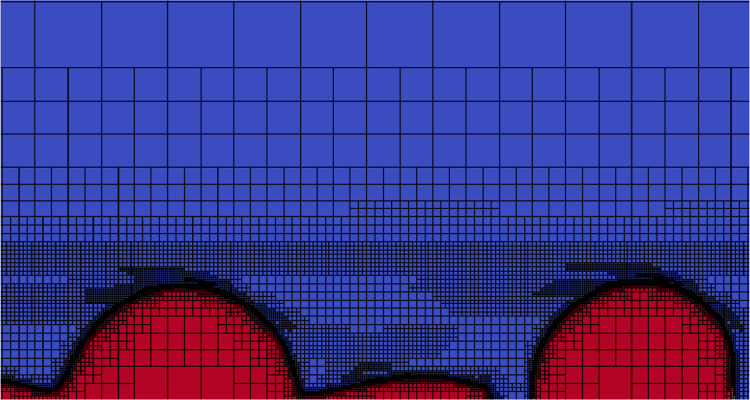
Example of an adaptative mesh with a maximum level of refinement 15; the liquid is in red and the air in blue.

With a refinement level of 15 in the most refined zone, the element size can be as small as $\Delta_{x}\approx 0.0156$ or approximately 1.5% of the initial jet radius. An example of the resulting adaptative mesh is plotted Fig. 5.

It is worth pointing out that the timestep is automatically adjusted so that the Courant number remains lower than 0.4.

### 2.3 Comparison with experimental data

**Fig. 6:**
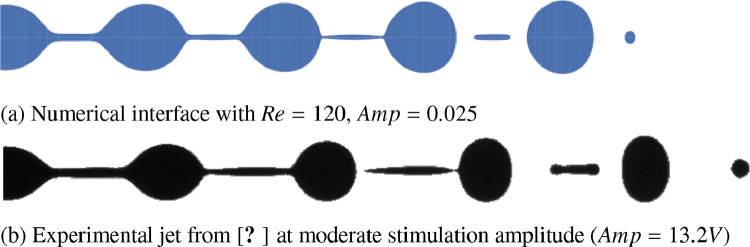
Comparison of jets interfaces obtained numerically (a) and experimentally (b) for Oh=0.2 and moderate stimulation amplitude.

The present numerical results is compared Fig. 6 to experimental ones from [8]. The experimental results have an Ohnesorge number $Oh=\frac{\sqrt{We}}{Re}=0.2$, corresponding to Re=120 in the present dataset. Although the numerical inlet velocity is different from the experimental one, it has been showed in [8], [9] that, until a moderate amplitude of stimulation, the jet morphology is not influenced by the nozzle geometry.

Both morphologies show a very good overall agreement and the small discrepancy observed is due to the different wave number x,

(7) $$x=\frac{2\pi R_{0}f}{u}$$

with x=0.9 and x=0.6 for the numerical and experimental jet, respectively.

### 2.4 Data collection

**Fig. 7:**
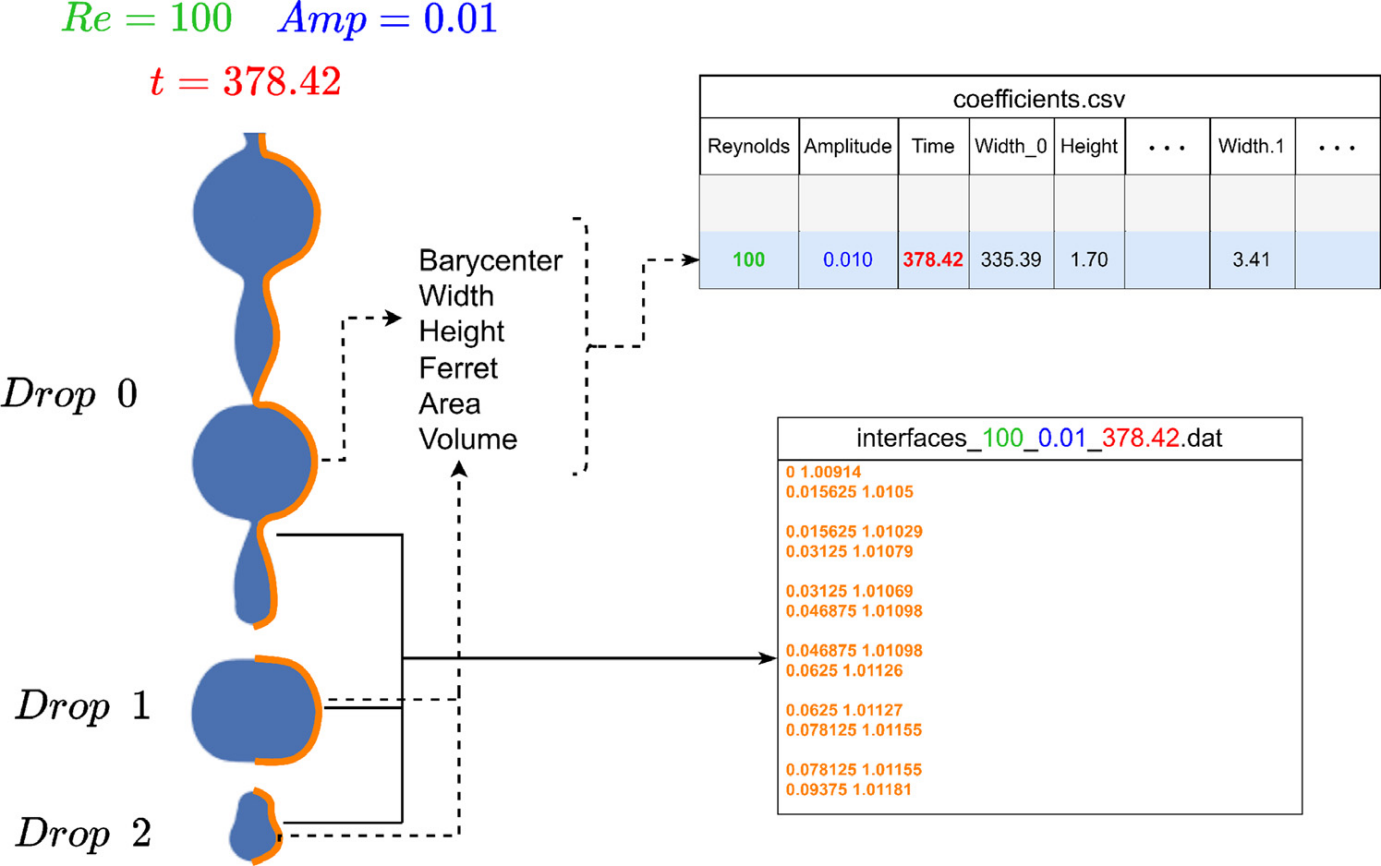
Overview of the generated data at simulation time t: both drop descriptors and fluid-air interface are generated and collected. An example is given for Re=100, Amp=0.010 and t=378.42.

A general overview of the methodology used to collect and generate the data is given Fig. 7.

For each simulation output, i.e. for each Re−Amp pair and at each simulation time t, 2 sets of information are generated:

1. descriptors of each drop are computed (area, volume, etc) and stored in *coefficients.csv*;
2. fluid-air interface segments are extracted and saved in *interfaces_Re_Amp_Time.dat* file.

## Ethics Statement

The authors followed generally expected standards of ethical behavior in scientific publishing throughout article construction.

## CRediT authorship contribution statement

**Guillaume Maîtrejean:** Conceptualization, Methodology, Software, Funding acquisition, Writing – original draft. **Adeline Samson:** Supervision, Conceptualization, Writing – review & editing, Project administration. **Denis C.D. Roux:** Formal analysis, Writing – review & editing.

## Declaration of Competing Interest

The authors declare that they have no known competing financial interests or personal relationships which have, or could be perceived to have, influenced the work reported in this article.

## Data Availability

Numerically-generated interfaces of Newtonian jets in CIJ regime (Original data) (Mendeley Data).
